# Agreement Between the European Organization for Research and Treatment of Cancer and Positron Emission Tomography Response Criteria in Solid Tumors in Evaluating Treatment Response in Solid Malignant Tumors

**DOI:** 10.7759/cureus.5422

**Published:** 2019-08-18

**Authors:** Zafar Nasir, Tariq Mahmood, Hatem Adel, Sadaf Nausheen, Samar Hamid, Amjad Sattar, Murli Manohar

**Affiliations:** 1 Radiology, Altnagelvin Area Hospital, Londonderry, GBR; 2 Radiology, Jinnah Post Graduate Medical Centre, Karachi, PAK; 3 Radiology, Dow University of Health Sciences, Karachi, PAK; 4 Radiology, Jinnah Postgraduate Medical Center, Karachi, PAK

**Keywords:** solid malignant tumors, response evaluation, radiological tumor response

## Abstract

Introduction

Fluorine-18 fluorodeoxyglucose (FDG) positron emission tomography-computed tomography (PET-CT) is used for non-invasive staging and restaging of solid malignant tumors. PET-CT based criteria have been developed to evaluate the response to targeted therapy. These include the European Organization for Research and Treatment of Cancer (EORTC) and the PET Response Criteria in Solid Tumors (PERCIST). The aim of this study was to determine the agreement between EORTC and PERCIST criteria for treatment response evaluation in patients with solid malignant tumors.

Materials and methods

This was a retrospective study conducted from February 2017 till July 2017. Electronic medical records of patients diagnosed with solid malignant tumors were searched. Experienced radiologists evaluated the PET-CT images based on EORTC and PERCIST criteria. The Kappa (κ) test was used for evaluation of agreement between treatment response according to EORTC and PERCIST criteria.

Results

Out of 54 patients, 41 (75.9%) were male and 13 (24.1%) were female with a mean age of 57.09 ± 10.65 years. According to EORTC criteria, complete metabolic response (CMR) was seen in five (9.3%) of patients, partial metabolic response (PMR) was seen in 36 (66.7%) of patients, progressive metabolic disease (PMD) was seen in nine (16.7%) of patients and stable metabolic disease (SMD) was seen in four (7.4%) of patients. According to PERCIST criteria, CMR was seen in five (9.3%) of patients, PMR was seen in 33 (61.1%) of patients, PMD was seen in nine (16.7%) of patients and SMD was seen in seven (13.0%) of patients. EORTC and PERCIST agreed on 43 (79.6%) of the patients with κ-coefficient of 0.62 indicating good agreement (p-value of <0.001).

Conclusion

EORTC and PERCIST criteria have a good agreement in evaluating treatment response in solid malignant tumors. Therefore, adoption of EORTC or PERCIST in PET-CT reporting can standardize the evaluation of oncological treatment results.

## Introduction

Fluorine-18 fluorodeoxyglucose (FDG) positron emission tomography-computed tomography (PET-CT) is an imaging modality for non-invasive staging and restaging of solid malignant tumors. The evaluation of oncological treatment response is based on changes in the number of malignant cells following therapy [1]. FDG PET-CT is used for preoperative staging of disease processes as well as for detection of recurrence [2-3].

For evaluating treatment response, tissue examination is used [4]. However, obtaining tissue samples is not always possible, especially in patients in treatment or post-treatment because of performing re-invasion and repetitive painful procedure. Moreover, bias also exists due to tumor heterogeneity [5]. Therefore, other techniques are often used for tumor response evaluation and imaging modalities provide a non-invasive means to evaluate them [6]. Imaging provides evaluation of anatomical, functional as well as molecular characteristics of tumors [7]. Moreover, a change in the size of the tumor can indicate the radiological tumor response [8].

Criteria for response evaluation have been developed for standardized evaluation of tumor treatment response. The commonly used criteria for solid tumor evaluation include WHO criteria and Response Evaluation Criteria in Solid Tumors (RECIST) [9]. PET-CT based criteria have also been developed to evaluate the response to targeted therapy [10]. Two criteria presently exist for PET-CT response evaluation; namely, European Organization for Research and Treatment of Cancer (EORTC), and PET Response Criteria in Solid Tumors (PERCIST) [11-12]. EORTC criteria are based upon the lesion-specific regions of interest (ROI) which are chosen at baseline and followed on subsequent scans [11]. However, in PERCIST criteria, a fixed ROI is drawn in the FDG-avid part of the lesion that is most metabolically active at each PET-CT examination.

EORTC and PERCIST have a different approach to tumor response evaluation. Therefore, this study was undertaken with the aim to determine the agreement between EORTC and PERCIST criteria for treatment response evaluation in patients with solid malignant tumors.

## Materials and methods

This was a retrospective study conducted at the Department of Radiology of Jinnah Postgraduate Medical Center (JPMC) from February 2017 till July 2017. The electronic medical records of patients diagnosed with solid malignant tumors were searched. Experienced radiologists evaluated the PET-CT images based on EORTC and PERCIST criteria. All PET-CT scans were performed on a 16 slicer PET-CT scanner (Celesteion, Toshiba Medical Systems, Tochigi, Japan). Patients fasted for six hours before the scan and were asked to maintain their hydration levels. Patients were injected with 3.7 MBq/kg 18F-FDG and the scan was performed 60 minutes after injection. Whole-body images were acquired from skull to mid-thigh. The scan parameters were as follows: 120 kVp, 20-200 mAs and 55 mm slice thickness. Following the acquisition of PET-CT, the images were sent to the workstation for analysis. According to EORTC criteria, the normalization of standardized uptake value (SUV) was done by the use of body surface area (BSA). BSA values were then calculated by the workstation automatically after the input of patient height and weight according to the formula of EORTC. In each study, the maximum SUV (SUVmax) of all targeted lesions was added and summed. For calculation of response evaluation, the smaller SUVmax was subtracted from the larger SUVmax and their difference was divided by the sum of SUVmax value from the initial scan. According to PERCIST criteria, the normalization of SUV was done by the use of lean body mass (LBM). It was calculated by the workstation upon the input of patient weight and height. Standardized uptake value normalized for lean body mass is referred to as SUL. For background activity assessment, a circular ROI of 3 cm was drawn on the right lobe of the liver, and in patients with liver involvement, the ROI was drawn on the descending aorta. The lesion having maximum SUL was identified and ROI of 1 cm was drawn on the part of the lesion having maximum activity. SUL value inside ROI was calculated as SULpeak. When evaluating treatment response, small SULpeak values were subtracted from larger SULpeak values. Their difference was divided by the sum of SULpeak value from the initial scan. Statistical analysis was carried out on the Statistical Package for Social Sciences v.22 (SPSS Inc., Chicago, Illinois, USA) version. Mean and standard deviation was calculated for age. Frequency and percentage were calculated for gender, tumor type, and responses by EORTC and PERCIST criteria. The Kappa (κ) test was used for evaluation of agreement between treatment response according to EORTC and PERCIST criteria. Values of 0.81 to 1.00 represented almost perfect agreement, between 0.61 to 0.80 represented good agreement, between 0.41 to 0.60 represented moderate agreement, between 0.21 to 0.40 represented fair agreement, and less than 0.20 represented poor agreement.

## Results

The study included 54 patients. The mean age of the patients was 57.09 ± 10.65 years. Around 41 (75.9%) were male and 13 (24.1%) were female. Among the solid malignant tumors, 17 (31.5%) were lung carcinoma, 16 (29.6%) were head and neck, 10 (18.5%) were colorectal carcinoma, seven (13.0%) were breast and four (7.4%) were gastric carcinoma. The mean follow-up duration was 3.3 ± 1.7 months. These are summarized in Table 1.

**Table 1:** Baseline characteristics of patients

Baseline characteristics of patients (n=54)			
	n	%	
Age, years	57.09 ±10.65^ǂ^		
Gender			
Male	41	75.9	
Female	13	24.1	
Type of solid malignant tumor			
Lung carcinoma	17		31.5
Head and neck carcinoma	16		29.6
Colorectal carcinoma	10		18.5
Breast carcinoma	7		13.0
Gastric carcinoma	4		7.4
^ǂ^mean±SD, n: number			

According to EORTC criteria, complete metabolic response (CMR) was seen in five (9.3%) of patients, partial metabolic response (PMR) was seen in 36 (66.7%) of patients, progressive metabolic disease (PMD) was seen in nine (16.7%) of patients and stable metabolic disease (SMD) was seen in four (7.4%) of patients.

According to PERCIST criteria, complete metabolic response (CMR) was seen in five (9.3%) of patients, partial metabolic response (PMR) was seen in 33 (61.1%) of patients, progressive metabolic disease (PMD) was seen in nine (16.7%) of patients and stable metabolic disease (SMD) was seen in seven (13.0%) of patients (Table 2).

**Table 2:** Agreement between EORTC and PERCIST criteria EORTC: European Organization for Research and Treatment of Cancer; PERCIST: PET Response Criteria in Solid Tumors; CMR: complete metabolic response; PMD: progressive metabolic disease; PMR: partial metabolic response; SMD: stable metabolic disease; k - kappa

Agreement between EORTC and PERCIST (n=54)					
Response with EORTC	Response with PERCIST				Total
	CMR	PMD	PMR	SMD	
CMR	5	0	0	0	5
PMD	0	9	0	0	9
PMR	0	0	29	7	36
SMD	0	0	4	0	4
κ-coefficient = 0.62 (p-value <0.001)					

EORTC and PERCIST agreed on 43 (79.6%) of the patients with κ-coefficient of 0.62 indicating good agreement (p-value of <0.001). EORTC and PERCIST disagreed on 11 (20.3%) patients.

## Discussion

In oncological care of patients, treatment response monitoring is of prime importance to determine the clinical prognosis of tumors in terms of response to treatment. Treatment response criteria have been developed to standardize the evaluation of treatment results. World Health Organization (WHO) and Response Evaluation Criteria in Solid Tumors (RECIST) were the first among these criteria evaluating the response by the size of the lesion [9]. With time, there has been an increase in the use of 18F-FDG PET-CT in oncology. Therefore, to standardize the response evaluation based on PET-CT, EORTC and PERCIST were developed [11-12]. Guidelines of EORTC recommend SUV normalization based on BSA. However, this criterion does not specify the number of lesions that should be measured or the minimum measurable lesion SUVmax [11]. In PERCIST criteria, LBM has been recommended for normalization of SUV. Moreover, the number of lesions to be measured, the minimum activity of the lesion to be measured, and features in two studies undergoing comparison were discussed in detail [12]. Therefore, it is of prime importance to be familiar with the similarities as well as the differences between EORTC and PET-CT response evaluation criteria. In the current study, the agreement between EORTC and PERCIST criteria was determined to evaluate treatment response in solid malignant tumors.

The results of our study have shown that there is a good agreement between EORTC and PERCIST criteria in evaluating treatment response to solid malignant tumors. However, another study done on solid malignant tumors has shown almost perfect agreement between these two criteria [13]. A potential reason for this could be a smaller sample size and difference in the types of solid malignant tumors in our population. In our study population, majority patients suffered from lung cancer followed by head and neck cancer, whereas in the previous study, colon and lung cancer was responsible for most of the cancer cases [13]. Another study focusing on colorectal cancer treatment response after irinotecan and cetuximab has shown good agreement between PERCIST and EORTC criteria [14], a finding that is comparable to our results.

A gap of almost ten years exists between publication of EORTC and PERCIST criteria. The research undertaken in this time period has allowed the approach to be more concise in the management of oncological patients. Both criteria take into account the complete patient, but from different perspectives. Quantification of metabolism and size of the tumor is embraced by EORTC criteria, whereas the most metabolically active part of the lesion describes almost the entire disease of the patient in PERCIST criteria.

Our study had several limitations. The consensus regarding the reading was not established. Interobserver agreement can help identify the variability, however, it was not studied. Another limitation of our study was that its results could not be generalized to all solid malignant tumors in our population due to a limited amount of solid malignant tumors in our study. Another limitation was that overall survival (OS) was not evaluated. Another limitation was that histopathology subtypes of tumors were not studied; different cell types have different uptake values and this can have an impact on treatment response. Moreover, as a modality, PET-CT cannot pick up brain lesions due to high physiologic uptake so in staging and evaluating treatment response in tumors especially lung and gastrointestinal tumors, brain metastasis requires special consideration for reflecting an accurate response to treatment in some cases.

These limitations aside, we believe that this study provides an insight regarding the use of PET-CT response criteria in a developing country. It is recommended that further studies should be carried out by increasing the number of observers as well as by evaluating the OS to study the impact of these criteria. Multicentric and prospective studies are recommended involving centers dealing in specialties where chances of obtaining multiple solid tumors are higher. Moreover, response evaluation of malignant tumors of other organ systems such as urogenital and central nervous systems should also be carried out.

## Conclusions

EORTC and PERCIST agreed on 76.9% of the patients with κ-coefficient of 0.62, indicating a good agreement in evaluating treatment response in solid malignant tumors. Therefore, adoption of EORTC or PERCIST in PET-CT reporting can standardize the evaluation of oncological treatment results.

## References

[1] Tan DS, Thomas GV, Garrett MD, Banerji U, de Bono JS, Kaye SB, Workman P (2009). Biomarker-driven early clinical trials in oncology: a paradigm shift in drug development. Cancer J.

[2] Figueiras RG, Goh V, Padhani AR (2010). Naveira AB, Caamano AG, Martin CV. The role of functional imaging in colorectal cancer. AJR Am J Roentgenol.

[3] Herbertson RA, Scarsbrook AF, Lee ST, Tebbutt N, Scott AM (2009). Established, emerging and future roles of PET/CT in the management of colorectal cancer. Clin Radiol.

[4] Yaghmai V, Miller FH, Rezai P, Benson III AB, Salem R (2011). Response to treatment series: part 2, tumor response assessment—using new and conventional criteria. AJR Am J Roentgenol.

[5] Fletcher JW, Djulbegovic B, Soares HP (2008). Recommendations on the use of 18F-FDG PET in oncology. J Nucl Med.

[6] Jaffe CC (2006). Measures of response: RECIST, WHO, and new alternatives. J Clin Oncol.

[7] Curran SD, Muellner AU, Schwartz LH (2006). Imaging response assessment in oncology. Cancer Imaging.

[8] Saini S (2001). Radiologic measurement of tumor size in clinical trials: past, present, and future. Am J Roentgenol.

[9] Shanbhogue AK, Karnad AB, Prasad SR (2010). Tumor response evaluation in oncology: current update. J Comput Assist Tomogr.

[10] Desar IM, van Herpen CM, van Laarhoven HW, Barentsz JO, Oyen WJ, van der Graaf WT (2009). Beyond RECIST: molecular and functional imaging techniques for evaluation of response to targeted therapy. Cancer Treat Rev.

[11] Young H, Baum R, Cremerius U (1999). Measurement of clinical and subclinical tumour response using [18F]-fluorodeoxyglucose and positron emission tomography: review and 1999 EORTC recommendations. European Organization for Research and Treatment of Cancer (EORTC) PET Study Group. Eur J Cancer.

[12] Wahl RL, Jacene H, Kasamon Y, Lodge MA (2009). From RECIST to PERCIST: evolving considerations for PET response criteria in solid tumors. J Nucl Med.

[13] Aras M, Erdil TY, Dane F (2016). Comparison of WHO, RECIST 1.1, EORTC, and PERCIST criteria in the evaluation of treatment response in malignant solid tumors. Nucl Med Commun.

[14] Skougaard K, Nielsen D, Jensen BV, Hendel HW (2013). Comparison of EORTC criteria and PERCIST for PET/CT response evaluation of patients with metastatic colorectal cancer treated with irinotecan and cetuximab. J Nucl Med.

